# Temporary Epicardial Pacing Wire Migration to the Pulmonary Artery in the Early Postoperative Stage

**DOI:** 10.7759/cureus.56446

**Published:** 2024-03-19

**Authors:** Soichiro Kageyama, Takeki Ohashi, Akinori Kojima

**Affiliations:** 1 Cardiovascular Surgery, Nagoya Tokushukai General Hospital, Kasugai, JPN

**Keywords:** acute type a aortic dissection, surgery, catheter therapy, residual wire migration, temporary epicardial pacing heart wires

## Abstract

Temporary epicardial pacing wires (TEPWs) are widely used during open heart surgery to treat postoperative bradycardia or arrhythmia. They are usually removed, but the wire is cut at the skin entrance site if there is resistance upon removal. Residual TEPWs have risks of complications such as infection, but they rarely migrate to distant organs. We report a case of TEPW migration from the right ventricle to the pulmonary artery during the early stage after acute type A aortic dissection surgery. Residual TEPW migration was detected incidentally during follow-up imaging for aortic dissection, and no other complications, such as residual wire infection or thrombus, were noted. The residual TEPW was safely treated using catheter therapy. This case report utilized existing patient information without intervention for research purposes, and the requirement for obtaining written patient consent was waived.

## Introduction

Temporary epicardial pacing wires (TEPWs) are widely used in open-heart surgery due to the development of bradycardia or arrhythmia. However, the pacing wire is often cut at the skin entrance site to minimize the potential of developing cardiac tamponade if there is resistance at the time of wire removal; cardiac tamponade is a major complication of pacing wire removal because of damage to the myocardium or coronary artery bypass grafting (CABG) [1-3]. Residual wires are associated with the risk of infection or migration, although they only occasionally migrate to remote organs. Here, we report a case of residual epicardial pacing heart-wire migration to the pulmonary artery during the early postoperative stage. This situation occurred approximately three months after an acute type A aortic dissection (AAAD) was treated with catheter therapy.

## Case presentation

A 51-year-old man with a sudden onset of chest pain and vital signs of shock was referred to our hospital. He was diagnosed with acute myocardial infarction (AMI) due to AAAD based on contrast-enhanced computed tomography (CT) findings, and emergency open-heart surgery was performed. Intimal entry tears were found in the aortic arch and root involving the right coronary artery (RCA). Partial root, ascending, and total arch replacements with a frozen elephant trunk and CABG procedures were performed.

Due to the AMI caused by RCA occlusion associated with aortic dissection, the patient had bradycardia after weaning from the cardiopulmonary bypass, and an OSYPKA heart wire type T (OSYPKA, Rheinfelden-Herten, Germany) was placed in the right ventricle.

**Figure 1:**
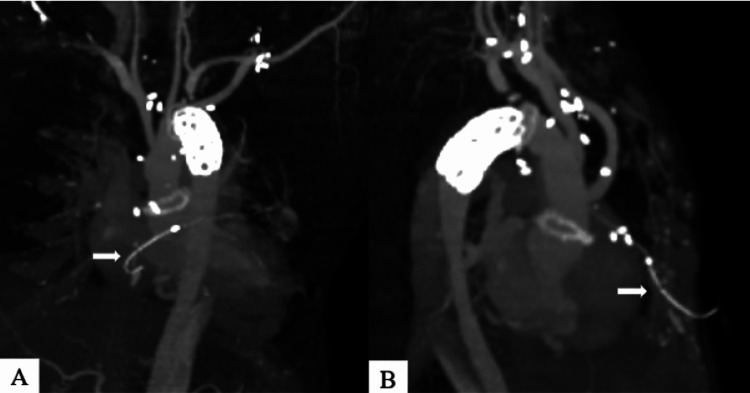
Computed tomography scan on postoperative day 8. (A) The residual TEPW remained near the skin at the anterior surface of the right ventricle (white arrow: residual TEPW). (B) Another view in the same computed tomography as (A) (white arrow: residual TEPW). TEPW: Temporary epicardial pacing wire

The postoperative course was uneventful, and the patient was extubated on postoperative day 2. On postoperative day 7, the TEPW was removed; however, resistance was encountered. To prevent potential damage to the CABG graft, the TEPW was cut at the skin entrance site. On postoperative day 8, contrast-enhanced CT revealed a patent CABG graft and no cardiac tamponade. The residual TEPW remained near the skin at the anterior surface of the right ventricle (Figure 1).

**Figure 2:**
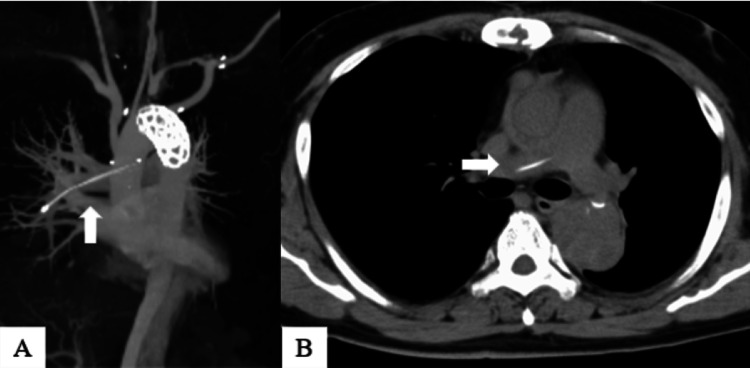
Computed tomography scan before additional thoracic endovascular aortic repair. (A) White arrow: residual TEPW migration to the right pulmonary artery. (B) Another view in the same computed tomography as (A) (white arrow: residual TEPW in the right pulmonary artery). TEPW: Temporary epicardial pacing wire

After discharge from our hospital, the patient was followed up at an outpatient hospital. Due to the rapid enlargement of the dissecting aortic aneurysm and back pain, additional thoracic endovascular aortic repair (TEVAR) was performed 124 days after the initial surgery. CT before the additional TEVAR showed a linear, high-density body in the right pulmonary artery (Figure 2).

This was considered to be the TEPW that had migrated to the pulmonary artery. No thrombus was noted around the wire on enhanced CT imaging, and there was no significant elevation of the concentration of serum C-reactive protein or fever. The patient had persistent back pain and underwent TEVAR. After the general condition of the patient was stabilized, residual TEPW removal was performed 135 days after the initial open-heart surgery.

**Figure 3:**
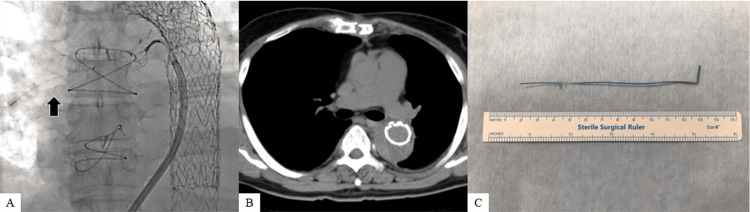
Catheter therapy to remove the TEPW. (A) Intraoperative finding (Black arrow: residual TEPW in the right pulmonary artery). (B) Computed tomography after catheter therapy. There is no residual wire in the right pulmonary artery. (C) Removed residual TEPW. TEPW: Temporary epicardial pacing wire

An Agilis™ NxT steerable sheath (Abbott, Chicago, IL, USA) was inserted from the femoral vein and advanced into the pulmonary artery with the ablation catheter. The TEPW was removed using a snare. Postoperative CT revealed no residual TEPW in the pulmonary artery, and the patient was discharged after an uneventful postoperative course. The patient is currently undergoing outpatient follow-up (Figure 3).

## Discussion

TEPWs are commonly used in open-heart surgery and manually removed when no longer required. However, the lead is often cut at the skin entrance site to prevent myocardial damage or CABG graft injury if there is resistance during wire removal [1-3]. Residual TEPW migration has been reported in the right heart system, as reported in this case. It has also been observed in remote organs, such as the ascending aorta, left ventricle, sternum, mandible, and abdominal organs, although that is extremely rare [1-9]. Usually, it is asymptomatic and discovered incidentally [1-3].

The mechanism underlying residual TEPW migration remains unclear [1,5]. Several reports have suggested that residual TEPW migration occurs due to cardiac pulsation, and a similar mechanism could be inferred for the present case.

**Figure 4:**
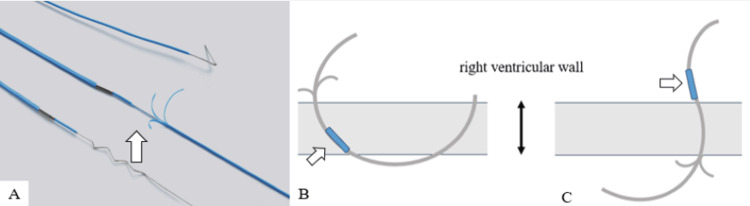
Predictive schematic diagram of TEPW movement in this case. (A) White arrow: OSYPKA heart wire type T. (B) Schematic diagram of TEPW penetration through all layers of the right ventricular wall (white arrow: electrode). (C) Schematic diagram of the TEPW tip dropped out into the right ventricular cavity (white arrow: electrode). TEPW: Temporary epicardial pacing wire Image Credits: Figure 4 has been created by Soichiro Kageyama M.D.

The OSYPKA type T heart wire used in the present case was fixed to the myocardium with a small portion at the tip of the electrode (Figure 4A). Ligature fixation is usually prohibited to prevent difficult removal; however, bleeding was observed at the wire entrance site in the present case, and suture hemostasis was performed. However, the wire, which was eventually removed from the pulmonary artery, was bent at 90° immediately below the resection point. It was inferred that bending of the wire occurred when resistance was applied. Thus, the wire was considered to have been caught in the hard tissue near the skin entrance site, such as the sternum or rectus abdominis fascia (Figure 3C). Ligature fixation to the myocardium did not appear to be the cause of difficult removal, but bleeding at the wire entrance site suggested that the wire penetrated all myocardial layers of the right ventricle. The CT scan after the TEPW was cut showed that the electrode had moved to the right ventricular surface (Figure 1B), and if the TEPW had been implanted through all layers of the right ventricle wall, the tip, including the tined part, could have dropped into the right ventricle cavity (Figures 4B, 4C). It is hypothesized that cardiac pulsation causes migration of the residual TEPW from the right ventricle to the pulmonary artery.

It has been reported that the removal of the residual TEPW using a snare catheter is effective [3,5,8,9]. Some cases require reopening of the chest and extracorporeal circulation to remove the residual TEPW [10].

## Conclusions

Complications from residual TEPWs are rare, but previous reports have argued for total removal of the wire. In our case, it is not clear whether the heart wire penetrated all layers of the right ventricular wall. However, the wire should be removed and placed at another location if there is bleeding at the TEPW entrance site. If the TEPW cannot be completely removed, the patient should be carefully followed up for complications such as residual TEPW migration.
